# Motor Deficits and Decreased Striatal Dopamine Receptor 2 Binding Activity in the Striatum-Specific *Dyt1* Conditional Knockout Mice

**DOI:** 10.1371/journal.pone.0024539

**Published:** 2011-09-12

**Authors:** Fumiaki Yokoi, Mai Tu Dang, Jianyong Li, David G. Standaert, Yuqing Li

**Affiliations:** 1 Department of Neurology, College of Medicine, University of Florida, Gainesville, Florida, United States of America; 2 The Children's Hospital of Philadelphia, Philadelphia, Pennsylvania, United States of America; 3 Department of Biochemistry, Virginia Tech, Blacksburg, Virginia, United States of America; 4 Department of Neurology, School of Medicine, Center for Neurodegeneration and Experimental Therapeutics, University of Alabama at Birmingham, Birmingham, Alabama, United States of America; Tokyo Medical and Dental University, Japan

## Abstract

DYT1 early-onset generalized dystonia is a hyperkinetic movement disorder caused by mutations in *DYT1* (*TOR1A*), which codes for torsinA. Recently, significant progress has been made in studying pathophysiology of DYT1 dystonia using targeted mouse models. *Dyt1* ΔGAG heterozygous knock-in (KI) and *Dyt1* knock-down (KD) mice exhibit motor deficits and alterations of striatal dopamine metabolisms, while *Dyt1* knockout (KO) and *Dyt1* ΔGAG homozygous KI mice show abnormal nuclear envelopes and neonatal lethality. However, it has not been clear whether motor deficits and striatal abnormality are caused by *Dyt1* mutation in the striatum itself or the end results of abnormal signals from other brain regions. To identify the brain region that contributes to these phenotypes, we made a striatum-specific *Dyt1* conditional knockout (*Dyt1* sKO) mouse. *Dyt1* sKO mice exhibited motor deficits and reduced striatal dopamine receptor 2 (D2R) binding activity, whereas they did not exhibit significant alteration of striatal monoamine contents. Furthermore, we also found normal nuclear envelope structure in striatal medium spiny neurons (MSNs) of an adult *Dyt1* sKO mouse and cerebral cortical neurons in cerebral cortex-specific *Dyt1* conditional knockout (*Dyt1* cKO) mice. The results suggest that the loss of striatal torsinA alone is sufficient to produce motor deficits, and that this effect may be mediated, at least in part, through changes in D2R function in the basal ganglia circuit.

## Introduction

Dystonia is a movement disorder that is exhibited by involuntary, repetitive, sustained muscle contractions or abnormal postures [1]. Dystonia is classified into two groups, primary and secondary dystonia. Primary dystonia develops spontaneously in the absence of any apparent cause or associated disease. Secondary dystonia is caused by other diseases, such as Parkinson's or Huntington's diseases, brain injury, or drug side effects. Genetic dystonia belongs to the primary dystonia group and is classified into more than 20 types, although less than half of them have known gene mutations [2]. DYT1 early-onset generalized torsion dystonia is an inherited movement disorder and is caused by mutations in *DYT1* (*TOR1A*) coding for torsinA with about 30% penetrance [3]. TorsinA is a member of AAA+ family of ATPases and may work in trafficking of polytopic membrane proteins and protein processing in the secretory pathway [4], [5]. ATPase activity [6], [7] and molecular chaperon activity of torsinA [8] were also reported *in vitro*. Nearly all DYT1 dystonia patients have a 3 base-pairs deletion (ΔGAG) in *DYT1* in one allele, corresponding to a loss of a glutamic acid residue in the C-terminal region of torsinA [3], while an 18 bp deletion mutation [9] and an Arg288Gln missense mutation [10] have been reported. A frame shift mutation caused by 4 bp-deletion coding for the C-terminal region of torsinA was also reported in a possible myoclonus-dystonia patient [11]. *Dyt1* ΔGAG heterozygous KI male mice exhibit reduction of striatal torsinA and motor deficits [12]–[14]. *Dyt1* KD male mice and *Dyt1* cKO mice also exhibit motor deficits, suggesting loss of torsinA function may contribute to motor deficits in these *Dyt1* mutant mice [15], [16]. Ampicillin-injected *Dyt1* ΔGAG heterozygous KI male mice express normal level of striatal torsinA and exhibit normal motor performance, suggesting recovery of torsinA level in the striatum may rescue the motor deficits in *Dyt1* ΔGAG heterozygous KI male mice [13].

Although DYT1 dystonia patients do not respond to levodopa treatment in most cases, there are several reports suggesting functional alterations in the striatal dopaminergic system in DYT1 dystonia. A postmortem dopamine (DA) content analysis in a DYT1 dystonia patient brain suggested a reduced DA in rostral portions of the putamen and caudate nucleus [17]. Another postmortem study reported a reduced dopamine receptor binding in the striatum and increased striatal 3,4-dihydroxyphenylacetic acid (DOPAC)/DA ratio in DYT1 dystonia patients [18]. A positron emission tomography study suggested that striatal D2R availability is reduced in both manifesting and non-manifesting *DYT1* mutation carriers [19]. *Dyt1* ΔGAG heterozygous KI male mice exhibit reduced homovanillic acid (HVA) level in the striatum [12] and *Dyt1* KD mice show decreased level of striatal DOPAC [15]. Moreover a transgenic mouse model overexpressing human mutant torsinA derived by human cytomegalovirus (CMV) immediate early promoter exhibit a reduction of D2R in the striatum [20]. These reports suggested that functional alterations of the striatal dopaminergic system may contribute to the pathogenesis of DYT1 dystonia, while its precise functional role has not been well defined. For example, it is not known whether motor deficits and alterations of the striatal dopaminergic system are caused by the mutation in the striatum itself or end results of abnormal signals from other brain regions.

Abnormal nuclear envelopes have been reported in transfected cells over-expressing the mutant forms of torsinA [21]–[23], *Dyt1* KO mice and *Dyt1* ΔGAG homozygous KI mice exhibiting neonatal lethality [24]. Both transgenic mice overexpressing human WT torsinA and mutant torsinA using murine prion promoter exhibit abnormal nuclear envelopes and abnormal motor performance [25]. It was suggested that neuron-specific nuclear envelope abnormality in *Dyt1* ΔGAG homozygous KI mice is caused by malfunction of torsinA with incomplete compensation by torsinB which is weakly expressed in neurons [26]. However, such abnormality has not been found in *Dyt1* ΔGAG heterozygous KI mouse or DYT1 dystonia patient brains, casting doubts about its role in the pathogenesis of DYT1 dystonia.

In the present study, we hypothesized that a selective loss of torsinA function in the striatum may affect motor performance in mice. We used *cre-loxP* technology [27] applied to mouse gene recombination [28] to selectively inactivate *Dyt1* in the striatum using a *Rgs9-cre* line that has restricted recombination in the striatum [29]. *Dyt1* sKO mice were made and their motor performance, dopaminergic system, and the role of torsinA in maintaining the nuclear envelope structures were analyzed. Since abnormal nuclear envelope was reported in cerebral cortical neurons in *Dyt1* KO and *Dyt1* ΔGAG homozygous KI mice, we further analyzed nuclear envelopes in cerebral cortical neurons of *Dyt1* cKO mice [16].

## Results

### Making of *Dyt1* sKO mice

**Figure 1:**
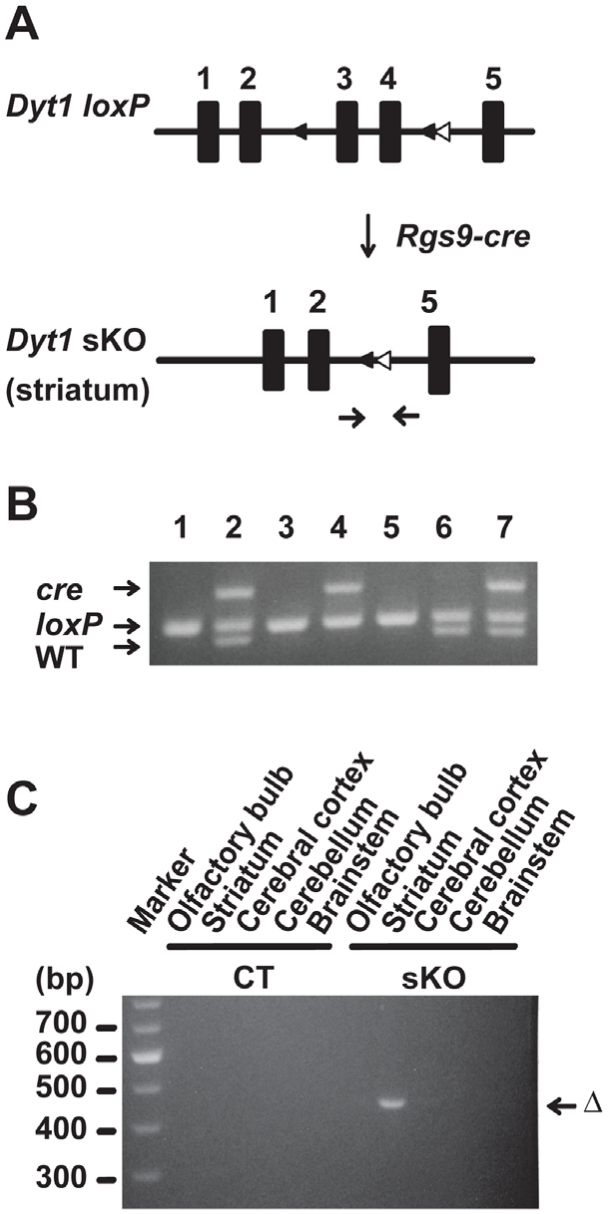
Making of *Dyt1* sKO mice. (A) The breeding strategy to generate *Dyt1* sKO mice. *Dyt1 loxP* mice [16] and *Rgs9-cre* mice [29] were prepared as described earlier. *Rgs9-cre Dyt1 loxP* double heterozygous mice were prepared by crossing *Dyt1 loxP* mice and *Rgs9-cre* mice. *Dyt1* sKO mice were prepared by crossing *Rgs9-cre Dyt1 loxP* double heterozygous mice and *Dyt1 loxP* mice. The primer sites to amplify *Dyt1* exons 3 and 4-deleted locus were shown by an arrow pair under the map. (B) A representative image of the multiplex PCR-based genotyping. Top bands are PCR products of *cre*. Middle bands are PCR products of *Dyt1 loxP* locus. The bottom bands are PCR products of *Dyt1* WT locus. Lanes 1, 3, 5: *Dyt1 loxP* homozygous mice; Lanes 2, 7: *Rgs9-cre Dyt1 loxP* double heterozygous mice; Lanes 4: *Dyt1* sKO mouse; Lane 6: *Dyt1 loxP* heterozygous mouse. (C) Tissue-specific deletion of *Dyt1* exons 3 and 4 in *Dyt1*sKO mice was confirmed by PCR using DNA isolated from each brain region. The deletion was detected only in the striatum of *Dyt1*sKO mouse as predicted (Δ). CT: control littermate mouse.

*Rgs9-cre* and *Dyt1 loxP* double heterozygous mice were prepared by crossing *Dyt1 loxP* mice [16] and *Rgs9-cre* mice [29]. *Dyt1* sKO mice were prepared by crossing *Rgs9-cre Dyt1 loxP* double heterozygous mice and *Dyt1 loxP* mice (Fig. 1A). Genotyping was performed by multiplex PCR with tail DNA (Fig. 1B). *Dyt1* sKO mice were born according to Mendelian ratio and developed to adult. The striatum-specific deletion of *Dyt1* exons 3 and 4 in *Dyt1* sKO mice was confirmed by PCR using DNA isolated from each brain region (Fig. 1C).

### No overt abnormal postures and normal locomotion in *Dyt1* sKO mice

When suspended from the tail, both *Dyt1* sKO and Control littermate (CT) mice showed normal splaying of hindpaws and had no observable hindpaw extension or truncal arching. All mice exhibited strong righting reflexes when tipped on their side. The results suggest that *Dyt1* sKO mice had no overt abnormal postures.

**Figure 2:**
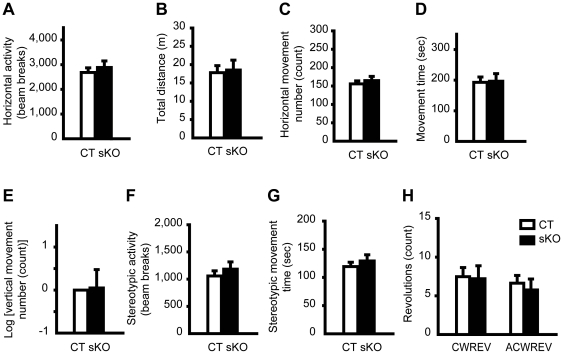
Locomotion of *Dyt1* sKO mice and CT mice in the open field test. Spontaneous activities in *Dyt1* sKO mice were assessed in the open-field apparatus and compared to those in CT mice. *Dyt1* sKO mice did not exhibit significant differences in comparison to CT mice in horizontal locomotion (A, horizontal activity; B, total distance; C, movement number; D, movement time) or vertical locomotion (E, vertical movement number). There was no significant difference in stereotypic activity (F), stereotypic movement time (G), clockwise (H, CWREV) or anti-clockwise revolution (H, ACWREV). Vertical bars represent means ± standard errors.

Since *Dyt1* ΔGAG heterozygous KI male mice [12] and *Dyt1* KD male mice [15] exhibit moderate hyperactivities, and *Dyt1* cKO mice exhibit prominent hyperactivities in the open-field test [16], spontaneous activities in *Dyt1* sKO mice were assessed in the open-field apparatus and compared to those in CT mice. *Dyt1* sKO mice did not exhibit significant differences in comparison to CT mice in either horizontal locomotion (Fig. 2A; horizontal activity, *p* = 0.55; B; total distance, *p* = 0.83; C; movement number, *p* = 0.54; D; movement time, *p* = 0.91) or vertical locomotion (Fig. 2E; vertical movement number, *p* = 0.87). There was no significant difference in stereotypic activity (Fig. 2F; *p* = 0.45), stereotypic movement time (Fig. 2G; *p* = 0.47), clockwise or anti-clockwise revolution between *Dyt1* sKO mice and CT mice (Fig. 2H; *p* = 0.90 and *p* = 0.62, respectively). The results suggest that loss of torsinA in the striatum alone does not affect spontaneous locomotion.

### Significant motor deficits of hindpaws in *Dyt1* sKO mice

**Figure 3:**
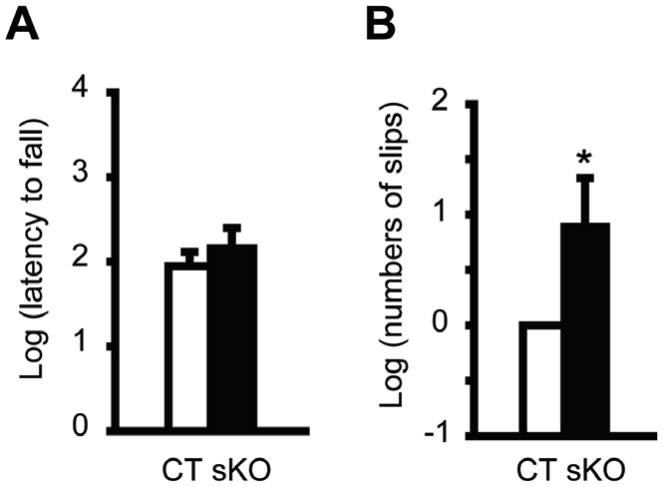
Motor performances in *Dyt1* sKO mice and CT mice. (A) Latency to fall in the accelerated rotarod test. *Dyt1* sKO mice did not exhibit significant difference in the latency to fall, suggesting no motor deficits with four paws. (B) Beam-walking performances in *Dyt1* sKO mice and CT mice. The data in the CT mice were normalized to zero. *Dyt1* sKO mice showed significant increased slips numbers in beam-walking test, suggesting motor deficits in hindpaws. Vertical bars represent means ± standard errors. *p<0.05.

Motor performance was assessed by the accelerated rotarod and beam-walking tests. Each mouse was put on the accelerated rotarod and the latency to fall was measured. Since mice can hold onto the rotarod with four paws, the latency to fall is an indicator of total motor performance and shorter latency indicates motor deficits. *Dyt1* sKO mice did not show significant difference in latency to fall (Fig. 3A; *p* = 0.48), suggesting no motor symptoms in total motor performance with four paws. We further analyzed the motor coordination and balance by the beam-walking test. Mice were trained to transverse a medium square beam for two days. The trained mice were tested twice on four different beams and total numbers of hindpaw slips were analyzed. *Dyt1* sKO mice showed 144% more slip numbers in the beam-walking test (Fig. 3B; *p* = 0.043), suggesting motor deficits in hindpaws.

In the accelerated rotarod test, *Dyt1* sKO mice did not exhibit overt motor deficits with four paws. The result is consistent with other DYT1 dystonia mouse models. *Dyt1* ΔGAG heterozygous KI mice [12], *Dyt1* KD mice [15] or *Dyt1* cKO mice [16] do not exhibit significant motor deficits either in the accelerated rotarod tests. On the other hand, *Dyt1* sKO mice, *Dyt1* cKO mice, *Dyt1* ΔGAG heterozygous KI male mice and *Dyt1* KD male mice exhibit motor deficits in the beam-walking test. Since dystonic symptoms commonly start from the legs in DYT1 dystonia patients, the beam-walking test may be one of the most appropriate behavior tests to detect the early motor symptoms in DYT1dystonia mouse models.

### Decreased radioligand binding to D2R in the striatal membrane fractions from *Dyt1* sKO mice

**Figure 4:**
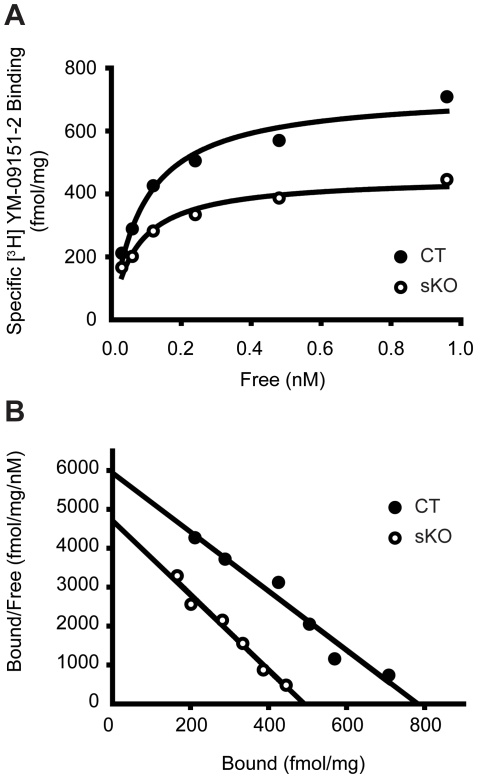
Radioligand binding activity to D2R in the striatal membrane fractions. (A) A representative saturation binding curve of [^3^H] YM-09151-2 to the striatal membranes fractions from three *Dyt1* sKO (open circles) and three CT (solid circles) mice. The transformed composite data of mean values obtained for the three mice in each genotype are plotted. (B) A representative Scatchard transformation of the data. The fit lines were created by least square means from the transformed composite data of mean values.

We measured radioligand binding activity of [^3^H] YM-09151-2 to D2R in the striatal membrane fractions from three *Dyt1* sKO and three CT mice. To see the overall distribution of data, representative saturation binding curves of [^3^H] YM-09151-2 were drawn using the transformed composite data of mean values obtained for the three *Dyt1* sKO (open circles) and three CT (solid circles) mice (Fig. 4A). The fit lines in the representative Scatchard plot were also created by least square means from the transformed composite data of the mean values (Fig. 4B; *Dyt1* sKO, r^2^ = 0.9788; CT, r^2^ = 0.9585) as described [30]. To analyze the statistical significance of *B_max_* and *K_d_*, between *Dyt1* sKO and CT mice, *B_max_* and *K_d_* of each mouse were also individually analyzed in Scatchard plot and those of each genotype were compared by Student's *t* test as described [31]. The *B_max_* value for [^3^H] YM-09151-2 binding, reflecting levels of radioligand binding to D2R, was significantly lower in *Dyt1* sKO mice in comparison to CT mice (means ± standard errors; *Dyt1* sKO, 541±28 fmol/mg, n = 3; CT, 793±46 fmol/mg, n = 3; *p* = 0.009). Receptor affinity for the ligand as indicated by *K_d_*, however, was unchanged in *Dyt1* sKO mice (*Dyt1* sKO, 149± 46 pM, n = 3; CT, 135±12 pM, n = 3; *p* = 0.79).

### Normal monoamine contents in the striatum of *Dyt1* sKO mice

To determine whether the motor deficits accompany any alterations of striatal DA metabolism, we measured concentrations of DA, DOPAC and HVA in the striatum. Unlike *Dyt1* ΔGAG heterozygous KI mice or *Dyt1* KD mice, the levels of DA, DOPAC or HVA and the ratios of DOPAC or HVA to DA in the *Dyt1* sKO mice were not significantly different from those in CT mice (Table 1); suggesting loss of torsinA function in the striatum alone does not affect monoamine contents in the striatum.

**Table 1:** DA and its metabolites in the striatum.

Content or ratio	CT	*Dyt1* sKO	*p*
**DA**	8.65±2.36	9.26±2.29	0.86
**DOPAC**	0.82±0.12	0.76±0.11	0.70
**HVA**	1.37±0.14	1.36±0.13	0.98
**DOPAC/DA**	2.58±0.21	2.51±0.20	0.82
**HVA/DA**	3.19±0.23	3.18±0.22	0.98

The values of neurochemical are shown as mean ± standard errors (in ng/mg of wet tissue). The turnovers of metabolites are shown as the ratio of the neurochemicals after natural log transformation to obtain a normal distribution. CT: control littermate mice; *Dyt1* sKO: striatum-specific *Dyt1* conditional knockout mice.

### Normal nuclear envelopes of neurons in *Dyt1* sKO and *Dyt1* cKO mice

**Figure 5:**
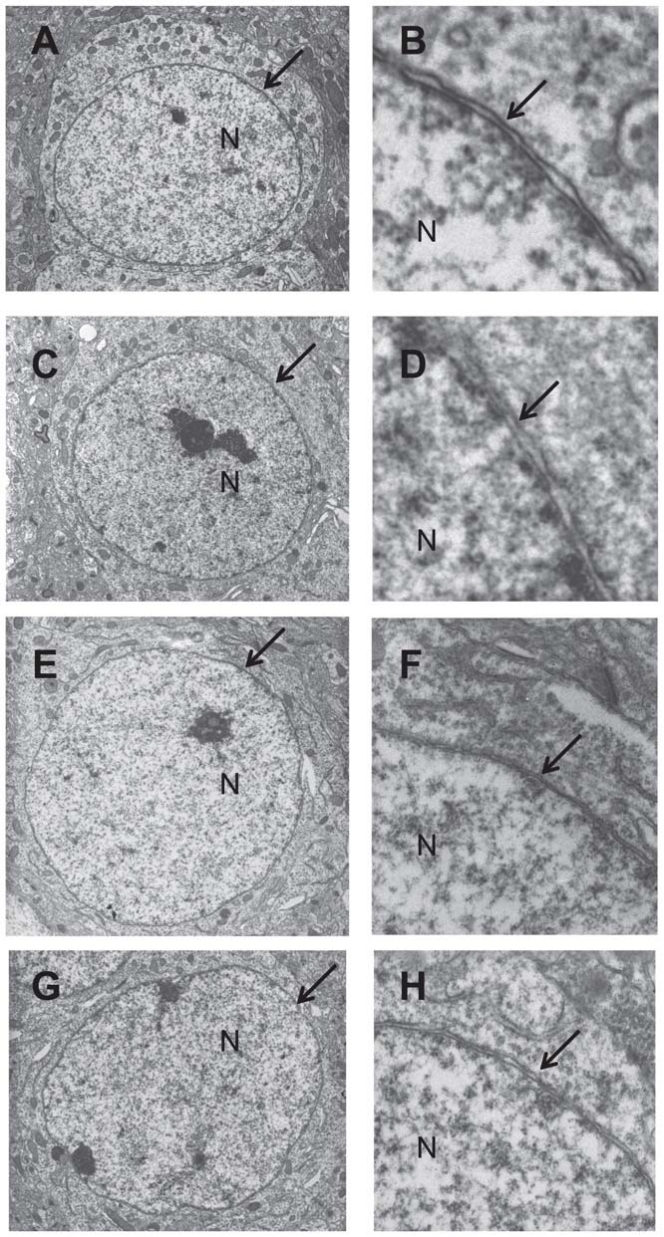
Normal nuclear envelope structure of the striatal MSNs in *Dyt1* sKO and CT mice, and cerebral cortical neurons in *Dyt1* cKO and CT mice. Representative electron microscope images of the nuclei of the striatal MSNs in CT (A) and *Dyt1* sKO mice (C). Enlarged images of CT (B) and *Dyt1* sKO (D) mice clearly show normal nuclear envelopes in MSNs. Representative electron microscope images of the nuclei of the cerebral cortical neurons in CT (E) and *Dyt1* cKO (G) mice. Enlarged images of CT (F) and *Dyt1* cKO (H) mice also show normal nuclear envelopes in cerebral cortical neurons. Nucleus (N) and nuclear envelope (arrow) are shown in each figure. No abnormal nuclear envelopes of neurons were detected in *Dyt1* sKO or *Dyt1* cKO mice as well as CT mice. Magnification in A, C, E, G: 5,000×.

**Figure 6:**
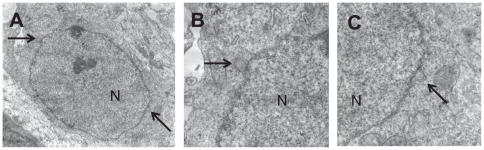
Abnormal nuclear envelope structure of the cerebral cortical neurons in a newborn *Dyt1* ΔGAG homozygous KI mouse. (A) A representative electron microscope image of the cerebral cortical neurons of a newborn *Dyt1* ΔGAG homozygous KI mouse. This section has two abnormal nuclear envelope structures (arrows) on the left and right parts of the image. Enlarged images of the left (B) and the right (C) parts of the nuclear envelope clearly show abnormal nuclear envelope structures in the cerebral cortical neuron. Nucleus (N) and abnormal nuclear envelope structure (arrow) are shown in each figure. Magnification in A: 6,000×.

To analyze whether loss of torsinA in adult neurons affects nuclear envelope structures, we examined the nuclear envelope structures of striatal MSNs in a *Dyt1* sKO mouse using a transmission electron microscopy. We first examined 11 striatal MSNs in a CT mouse and found no abnormal nuclear envelope as expected (Fig. 5A, B). Although we examined 29 striatal MSNs of a *Dyt1* sKO mouse, we could not find any blebbing or other nuclear envelope abnormalities among them (Fig. 5C, D). Since abnormal nuclear envelope was reported in cerebral cortical neurons of *Dyt1* KO and *Dyt1* ΔGAG homozygous KI mice but not in their striatal neurons [24], we further analyzed cerebral cortical neurons in *Dyt1* cKO mice to determine whether loss of torsinA in cerebral cortical neurons contributes to abnormal envelope in adult mice. Although we examined 50 cerebral cortical neurons of two CT mice (Fig. 5E, F) and 84 cerebral cortical neurons of two *Dyt1* cKO mice (Fig. 5G, H), we could not find any blebbing or other nuclear envelope abnormalities among them, either. Since *Dyt1* cKO mice exhibit motor deficits [16], these results suggest that abnormal nuclear envelope structure is not a cause of motor impairment in *Dyt1* cKO or sKO mice. On the other hand, we confirmed abnormal nuclear envelopes in the cerebral cortical neurons of our newborn *Dyt1* ΔGAG homozygous KI mouse [12] used as a positive control (Fig. 6A–C).

## Discussion

Although *DYT1* is expressed in multiple brain regions, motor symptoms are prominent in DYT1 dystonia patients. Reduced D2R binding activity and alteration of striatal monoamine metabolism were reported in DYT1 dystonia patients [17]–[19]. *Dyt1* ΔGAG heterozygous KI male and *Dyt1* KD male mice exhibit motor deficits. *Dyt1* ΔGAG heterozygous KI male mice exhibit reduced HVA in the striatum [12] and *Dyt1* KD mice exhibit reduced striatal DOPAC [15]. Furthermore, a transgenic mouse model overexpressing human mutant torsinA derived by human CMV immediate early promoter show reduction of D2R in the striatum and impaired LTD which is rescued by adenosine A2A receptor antagonism [20]. In the same mouse model, altered responses to D2R activation and N-type calcium currents in striatal cholinergic interneurons were reported [32]. Moreover anticholinergic drugs are quite effective in clinical practice [1]. To determine whether the alterations of the dopaminergic system are cell-autonomous, we made *Dyt1* sKO mice and analyzed their motor performance and dopaminergic system. *Dyt1* sKO mice exhibited motor deficits and reduced striatal D2R binding activity, whereas they did not exhibit significant alteration of striatal monoamine contents. MSNs in the indirect pathway and cholinergic interneurons express D2R and contribute to synaptic plasticity in the striatum [33], [34]. Reduction of D2R may affect signal transduction pathways in these neurons and affect basal ganglia circuit. Since the loss of D2R is known to cause motor deficits in mice [31], the present results suggest that the loss of striatal torsinA affects signal transduction pathway through D2R in the basal ganglia circuit and exhibit motor deficits.

Several studies suggest a reduction of striatal D2R binding activity in DYT1 dystonia patients. A postmortem study reported a trend of [^3^H] YM-09151-2 binding reduction in the striatum [18]. Another positron emission tomography study also suggested that the striatal D2R availability was reduced in both manifesting and non-manifesting *DYT1* mutation carriers [19]. However, the mechanism of the striatal D2R binding activity reduction has not been known. In the present study, we made *Dyt1* sKO mice and analyzed their D2R binding activity. *B_max_* value for [^3^H] YM-09151-2 binding activity to D2R was significantly lower in *Dyt1* sKO mice in comparison to CT mice, while the receptor affinity to the ligand as indicated by *K_d_*, was comparable between the genotypes. The results suggest that the number of functional D2R may be reduced in the striatal membrane fractions, while the affinity of the ligand itself is not largely altered. The results also suggest that reduced striatal D2R binding activity is not an end result caused by alterations of striatal monoamines, but it is caused by loss of torsinA function in the striatum itself. It was suggested that torsinA contributes to protein processing in the secretory pathway [5]. Over-expression of WT torsinA *in vitro* also inhibit trafficking of polytopic membrane proteins [4], suggesting proper level of torsinA protein is critical for normal protein maturation and trafficking. Deficient trafficking of D2R caused by loss of torsinA in the striatum may underlie decreased D2R-binding in the striatal membrane fractions.

The results suggest that the loss of torsinA function in the striatum itself contributes to the pathophysiology of DYT1 dystonia. Loss of striatal torsinA may affect signal transduction pathway through D2R in the basal ganglia circuit and exhibit motor deficits. This is consistent with the clinical observation that deep brain stimulation targeting GPi in the basal ganglia circuits is an effective surgical therapy for DYT1 dystonia [35]. Interestingly, *Dyt1* cKO mice also exhibit similar motor deficits without alteration of monoamine contents in the striatum [16]. Collectively, our results suggest that loss of torsinA function in the corticostriatal pathway may affect the basal ganglia circuits and contribute to motor impairment without alteration of DA metabolism itself. Further studies focusing on the corticostriatal pathway will elucidate details of the molecular mechanism of this disease.

A postmortem study reported increased striatal DOPAC/DA ratio in DYT1 dystonia patients [18]. *Dyt1* ΔGAG heterozygous KI male mice exhibit decreased HVA [12], while *Dyt1* KD mice exhibited decreased DOPAC [15] in their striata. However, the mechanism of the striatal monoamine alterations has not been clear. In the present study, *Dyt1* sKO mice did not exhibit alteration of DA, DOPAC or HVA contents in the striatum, suggesting that loss of torsinA function in the striatum itself may not affect monoamine contents in the striatum. The results also suggest that dopaminergic tone in the striatum may have a limited role in the pathogenesis of DYT1 dystonia because *Dyt1* sKO mice exhibited motor deficit without alteration of striatal monoamine contents. The results are consistent with the clinical observation that levodopa is not effective for DYT1 patients. The significant alterations in the striatal DA metabolites found in *Dyt1* ΔGAG heterozygous KI male mice and *Dyt1* KD mice may be caused by loss of torsinA function in dopaminergic neurons derived from the substantia nigra. Generating substantia nigra-specific *Dyt1* conditional KO mice in the future may further allow us to determine the origin of the alterations of DA metabolism observed in *Dyt1* ΔGAG heterozygous KI male mice and *Dyt1* KD mice.

Abnormal nuclear envelopes have been reported in transfected cells over-expressing the mutant forms of torsinA [21]–[23]. Abnormal nuclear envelopes were also reported in *Dyt1* KO mice and *Dyt1* ΔGAG homozygous KI mice [24]. However, abnormal nuclear envelopes were not found in *Dyt1* ΔGAG heterozygous KI mice which exhibit motor deficits, casting doubts about its role in the pathogenesis of DYT1 dystonia. To analyze whether the motor deficits in *Dyt1* sKO and cKO mice are caused by abnormal nuclear envelopes, we examined the nuclear envelopes of striatal MSNs or cortical neurons. However, we could not find any blebbing or other nuclear envelope abnormalities in both *Dyt1* sKO and cKO mice, suggesting that nuclear envelope abnormality may not play any role in motor impairment seen in these mice. Since abnormal envelopes *in vivo* are detected only in neonatal *Dyt1* ΔGAG homozygous KI mice and *Dyt1* KO mice, that exhibit neonatal lethality, the abnormal nuclear envelope may be an indicator of neuronal cell death in these dying mice.

## Materials and Methods

### Making of the striatum-specific *Dyt1* conditional knockout mice

*Dyt1 loxP* mice [16] and *Rgs9-cre* mice [29] were prepared as described earlier. Genotyping for *Dyt1* sKO and CT mice was performed by multiplex PCR using tail DNA with F (5′-ATTCAAAAATGTTGTCATAGCCAGG-3′) and T (5′-CTACAGTGACCTGAATCATGTGGC-3′) primer sets for *Dyt1 loxP* locus [16], and creA (5′-ATCTCCGGTATTGAAACTCCAGCGC-3′) and cre6 (5′-CACTCATGGAAAATAGCGATC-3′) primer sets for *cre* locus [36]. To confirm the striatum-specific deletion of *Dyt1* exons 3 and 4, the olfactory bulb, striatum, cerebral cortex, cerebellum, and brainstem were dissected from *Dyt1 loxP* and *Dyt1* sKO mouse brains. The tissues were digested with lysis buffer [100 mM Tris•Cl (pH 8.5), 5 mM EDTA•2Na, 0.2% SDS, 200 mM NaCl, 1 mM CaCl_2_, 0.1 mg/ml Proteinase K (Invitrogen)] at 55°C overnight. Each DNA was isolated by adding equal volume of isopropanol and then washing with 70% ethanol. The deletion of exons 3 and 4 was confirmed by PCR using F and Tcko2 (5′-CCATAGCTGGACCTGCAATTAAG-3′) primers as described earlier [16], [37]. A group consisted of 11 *Dyt1* sKO mice (7 males and 4 females) and 13 CT mice (6 males and 7 females) was used for the behavior tests. A pair of *Dyt1* sKO and CT mice was used to prepare brain sections for transmission electron microscopy analysis. Since onset of DYT1 dystonia is usually childhood or adolescence, we used adult mice in this study. All experiments were performed by investigators blind to the genotypes. Mice were housed under a 12 hours-light and 12 hours-dark cycle with access to food and water *ad libitum*. All experiments were carried out in compliance with the USPHS Guide for Care and Use of Laboratory Animals and approved by IACUC of University of Alabama at Birmingham with Animal Protocol Number 091008198.

### Preparation of *Dyt1* cKO mice and a *Dyt1* ΔGAG homozygous KI mouse

Two pairs of adult *Dyt1* cKO and CT mice were prepared and genotyped as described earlier [16]. A neonatal *Dyt1* ΔGAG homozygous KI mouse was prepared by crossing *Dyt1* ΔGAG heterozygous KI mice and genotyped as described earlier [12], [38]. These mice were used for transmission electron microscopy analysis.

### Behavioral semi-quantitative assessments of motor disorders

Behavioral semi-quantitative assessments of motor disorders were performed as described earlier [12], [39]. Mouse from 96 to 139-days old was placed on a table and assessments of hindpaw clasping, hindpaw dystonia, truncal dystonia and balance adjustments to a postural challenge were performed. The hindpaw clasping was assessed as hindpaw movements for postural adjustment and attempt to straighten up while the mouse was suspended by the mid-tail. The hindpaw dystonia was assessed as the increased spacing between the limbs, poor limb coordination, crouching posture and impairment of gait. Truncal dystonia was assessed as the flexed posture. Postural challenge was performed by flipping the mouse onto its back and the ease of righting was noted.

### Accelerated rotarod test

The motor performance was assessed with Economex accelerating rotarod (Columbus Instruments) as described earlier [12]. The apparatus started at an initial speed of 4 rpm. Rod speed was gradually accelerated at a rate of 0.2 rpm/s. The latency to fall was measured with a cutoff time of 2 min. Mice from 103 to 146-days old were tested for three trials on each day for 2 days. The trials within the same day were performed at approximately 1 hour intervals.

### Open-field test

The open-field test was performed under light condition as described earlier [40], [41]. Spontaneous activities of individual mice from 124 to 167 days old were recorded by infrared light beam sensors in a 41×41×31 cm acryl case for 15 min at 1 min intervals using DigiPro software (AccuScan Instruments).

### Beam-walking test

The beam-walking test was performed as described earlier [12], [15], [41], [42]. Briefly, the beam-walking test was performed within the last 8 hours of the light period after acclimation to a sound-attenuated testing room for 1 hour. The mice from 141 to 184 days old were trained to transverse a medium square beam (14 mm wide) in three consecutive trials each day for 2 days and tested twice each on the medium square beam and a medium round beam (17 mm diameter) on the third day. The mice were then tested twice each on a small round beam (10 mm diameter) and a small square beam (7 mm wide) on the fourth day. Their hind paw slips on each side during transverse on the 80 cm-length beams were counted.

### Radioligand binding assay to D2R in the striatal membrane fractions

Radioligand binding assay to the striatal D2R was performed by using [^3^H] YM-09151-2 based on a method reported earlier [43]. The striata were dissected from three *Dyt1* sKO mice and three CT mice of 268–321 days old (average 284 days old) and homogenized in nine volumes of ice-cold 50 mM Tris•Cl, 8 mM MgCl_2_, 5 mM EDTA•2Na, pH 7.1. Each homogenate was centrifuged at 18,000× g for 20 min at 4°C and the pellet was suspended in the same buffer and stored at −80°C. The protein concentration of the membrane preparation was determined by Bradford assay using bovine serum albumin as a standard. An aliquot of the homogenate was suspended in 200 µl of binding buffer [50 mM Tris•Cl, 120 mM NaCl, 5 mM KCl, 5 mM MgCl_2_, 1.5 mM CaCl_2_, 1 mM EDTA•2Na, 10 µM pargyline hydrochloride (Sigma-Aldrich) and 0.1% ascorbic acid, pH 7.4] with 0.03–0.96 nM [^3^H] YM-09151-2 (2.64TBq/mmol, Perkin Elmer). The reaction mixture was incubated at 25°C for 40 min, and then rapidly filtered under vacuum through glass microfibre GF/B filter (Whatman). The filter was then washed four times with 4 ml of the ice-cold binding buffer without the isotope and then dried. The radioactivity of the filters was measured in 6 ml of ScintiSafe Econo1 (Fisher Scientific) by a Beckman liquid scintillation counter. Non-specific binding of [^3^H] YM-09151-2 was measured in presence of 30 µM (S)-(-)-sulpiride (Sigma-Aldrich). The experiments were performed in duplicate.

### HPLC analysis

Striata were dissected from the brains of 13 *Dyt1* sKO and 13 CT mice from 302 to 345 days old and homogenized in ice-cold 0.2 N perchloric acid. The homogenate were centrifuged for 15 min at 15,000 × g at 4°C to remove debris. Twenty microliters of the supernatant representing 2 mg of tissue, was the applied to a C18C reverse phase HPLC column (Varian) connected to an ESA model 5200A electrochemical detector. DA, DOPAC and HVA were analyzed using a running buffer of 50 mM potassium phosphate buffer with 0.5 mM octyl sulfate (Sigma) and 8% acetonitrile as described earlier [12], [15], [41]. DA, DOPAC, and HVA were separated at 0.8 ml/min and quantified by comparing to the standard reagents (Sigma).

### Transmission electron microscopy analysis

Brain sections for transmission electron microscopy were prepared as described earlier [14]. *Dyt1* sKO and their CT mice (n = 1 each, 6 weeks of age), *Dyt1* cKO mice and their CT mice (n = 2 each, 8 weeks of age), and a newborn *Dyt1* ΔGAG homozygous KI mouse were perfused with chilled 0.1 M phosphate-buffered saline (pH 7.4) followed by Karnovsky's fixative in phosphate buffered 2% glutaraldehyde and 2.5% paraformaldehyde. The brains were dissected out and left in Karnovsky's fixative overnight. The tissue was then trimmed and washed in cacodylate buffer with no further additives. Microwave fixation was used with the secondary 2% osmium tetroxide fixative, followed by the addition of 3% potassium ferricyanide for 30 minutes. After washing with water, saturated uranyl acetate was added for en bloc staining. The tissue was dehydrated in a series of increasing concentrations of ethanol starting at 50%. Acetonitrile was used as the transition fluid between ethanol and the epoxy. Infiltration series was done with an epoxy mixture using the epon substitute Lx112. The resulting blocks were polymerized at 90°C overnight, trimmed with a razor blade, and ultrathin sectioned with diamond knives. Sections were then stained with uranyl acetate and lead citrate, and the nuclear envelopes in the striata of *Dyt1* sKO and their CT mice, and cerebral cortices of *Dyt1* cKO mice, their CT mice and a *Dyt1* ΔGAG homozygous KI mouse were examined or photographed with a Hitachi H600 transmission electron microscope.

### Statistics

Data in the open-field test, latency to fall in the accelerated rotarod, and HPLC were analyzed by ANOVA mixed model with SAS/STAT Analyst program (Version 9.1.3; SAS institute Inc. NC) as described earlier [12], [16], [41]. The turnovers of metabolites were analyzed as the ratio of the neurochemicals after natural log transformation to obtain a normal distribution. Slips numbers of hindpaws in beam-walking test were analyzed by logistic regression (GENMOD) with Poisson distribution using GEE model in the software. Sex, age and body weight were input as variables. Vertical movement numbers in the open-field test, latency to fall in the accelerated rotarod test, and slips numbers in the beam-walking test were analyzed after natural log transformation to obtain a normal distribution. The data in CT mice were normalized to zero. Radioligand binding data were analyzed by Student's *t* test. *B_max_* and *K_d_* of each mouse were individually calculated in Scatchard plot and those of each genotype were compared. Significance was assigned at *p*<0.05.
